# Intracellular ratiometric temperature sensing using fluorescent carbon dots†

**DOI:** 10.1039/c8na00255j

**Published:** 2018-12-10

**Authors:** Jun-Ray Macairan, Dilan B. Jaunky, Alisa Piekny, Rafik Naccache

**Affiliations:** Department of Chemistry and Biochemistry, Center for NanoScience Research, Concordia University Montreal QC Canada H4B 1R6 rafik.naccache@concordia.ca; Department of Biology, Center for Cellular Microscopy and Cell Imaging, Concordia University Montreal QC Canada H4B 1R6

## Abstract

Highly sensitive non-invasive temperature sensing is critical for studying fundamental biological processes and applications in medical diagnostics. Nanoscale-based thermometers are promising non-invasive probes for precise temperature sensing with subcellular resolution. However, many of these systems have limitations as they rely on fluorescence intensity changes, deconvolution of peaks, or the use of hybrid systems to measure thermal events. To address this, we developed a fluorescence-based ratiometric temperature sensing approach using carbon dots prepared *via* microwave synthesis. These dots possess dual fluorescence signatures in the blue and red regions of the spectrum. We observed a linear response as a function of temperature in the range of 5–60 °C with a thermal resolution of 0.048 K^−1^ and thermal sensitivity of 1.97% C^−1^. Temperature-dependent fluorescence was also observed in HeLa cancer cells over a range of 32–42 °C by monitoring changes in the red-to-blue fluorescence signatures. We demonstrate that the ratiometric approach is superior to intensity-based thermal sensing because it is independent of the intracellular concentration of the optical probe. These findings suggest that dual-emitting carbon dots can be an effective tool for *in vitro* and possibly *in vivo* fluorescence nanothermometry.

## Abstract 2

A self-referencing dual fluorescing carbon dot-based nanothermometer can ratiometrically sense thermal events in HeLa cells with very high sensitivity.

## Graphical abstract

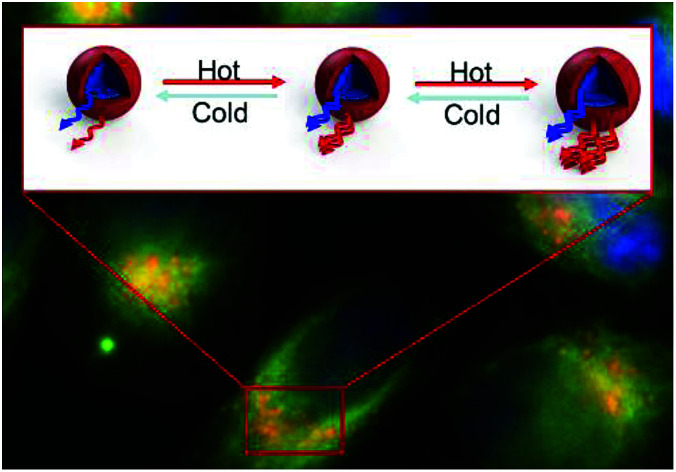

## Introduction

Temperature plays a vital role in many biological processes. Obtaining precise temperature readings in living organisms with high accuracy and specificity are crucial for fundamental cell studies, as well as for the development of novel diagnostic tools in nanomedicine.^1–5^ Recently, advancements in the field of nanotechnology have led to the development of novel sensing probes,^6–8^ which include nanothermometers (*i.e.* novel temperature sensors with high spatial resolution at the nanoscale).^4^ Such nanothermometers have been developed using different platforms ranging from fluorescent proteins to upconverting nanoparticles and quantum dots.^4,5,9–14^ Although still in the early stages of development, these luminescent nanomaterials have significant promise in temperature sensing and thermal mapping to grasp a better understanding of biological processes.^3–5,15^ The majority of reports concerning luminescent nanomaterials have focused on *in vitro* studies aimed at detecting the local temperature in a biological system at high resolution.^5,11,16–18^ This may potentially translate *in vivo* and could be useful as a diagnostic tool to detect inflammation and disease (*e.g.* cancer).^19,20^ Moreover, they could be used in synergy with other nanomaterials such as those studied for the development of novel hyperthermia treatments.^3,5^ In this case, combining thermal sensing with localized heating of the diseased area would minimize damage associated with overheating of the surrounding healthy tissue.^20–22^

Thermal sensing using nanomaterials can be accomplished by exploiting their optical properties. Fluorescence nanothermometry can be categorized into several classes depending on the specific parameter from which the thermal measurement is extracted including signal intensity, band-shape, fluorescence lifetimes, band-shift, excitation wavelength polarization and spectral shift, among others.^4^ To date, the most common fluorescent temperature-sensing probes extract temperature information by changes in signal intensity, variation in the fluorescence lifetimes, as well as band-shape.^3,5,9,14,15,23,24^ In the first case, fluorescence varies with changing temperature and may be observed as an absolute increase (or decrease) in signal. Lifetime nanothermometry is independent of signal intensity and allows for the extraction of thermal information through variation in the lifetime of the fluorescence event. Lastly, band-shape nanothermometry exploits the presence of two or more fluorescence bands. The fluorescence intensity (or area) is monitored for both bands over the temperature range and a comparative relationship is established. This method is advantageous over other methods since it is not concentration-dependent, does not rely on temperature-dependent decay curves and does not suffer from environmental interference (*e.g.* surrounding media or environment, variability in cell uptake at different temperatures, *etc.*).^18^

Among luminescent nanomaterials, carbon dots have garnered significant attention in recent years due to their versatile optical properties, notably tunable fluorescence and high photostability.^25–29^ In addition, they are dispersible in water, have excellent biocompatibility and low cytotoxicity.^25,29–34^ As such, they can be integrated into sensing, bioimaging and diagnostic applications such as fluorescent nanothermometry. Recently, carbon dot optical probes have been investigated for temperature sensing applications *in vivo* and *in vitro*.^9,16,23,24,35–37^ Yang *et al.*^24^ reported the synthesis of low cytotoxic biocompatible carbon dots with temperature-dependent fluorescence intensity from 20 to 80 °C and a thermal sensitivity of 0.85% °C^−1^. They extended their work to show temperature-dependent fluorescence in an *in vivo* mouse model. In another study,^16^ temperature-sensitive fluorescence lifetime carbon dot nanothermometers showed a sensitivity of 1.79% and promising results for temperature sensing in cells. In a different approach,^23^ a ratiometric carbon dot temperature sensing probe was developed over the range of 5 to 85 °C. This was achieved *via* deconvolution of overlapping fluorescence bands resulting in a thermal sensitivity of 1.48% °C^−1^. Wang *et al.*^38^ circumvented the need for deconvolution of peaks through the development of nanohybrids consisting of carbon dots and gold nanoclusters, which yield blue and red emission, respectively, with a thermal sensitivity of 1.8% °C^−1^.

Distinct fluorescent bands emanating from a single probe is ideal for ratiometric temperature sensing to minimize variability and ensure homogeneous physico-optical properties. Few reports have focused on dual-fluorescing carbon dots (dCDs)^23,39–41^ and the majority do not describe the use of these dots for fluorescence thermal sensing. To this end, we report a facile synthesis of biocompatible dCDs for fluorescence nanothermometry in live cells. Their capacity to simultaneously fluoresce in the blue and red regions of the spectrum originates from two different emissive states on the dCDs.^23,42^ Due to their unique optical properties, temperature sensing over the range of 5–60 °C can be accomplished *via* changes in fluorescence intensity or a ratiometric approach that utilizes both fluorescence signatures. The temperature sensitivity of the dCDs was assessed in colloidal dispersions and HeLa cancer cells, extending the thermometric relationship to biological systems.

## Results and discussion

### Physico-chemical and optical characterization

**Fig. 1:**
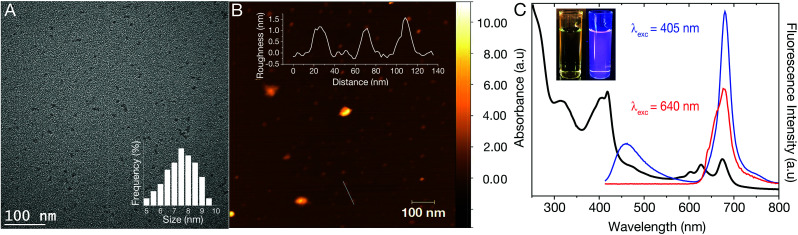
(A) TEM image of a 1 mg mL^−1^ dCD dispersion in water. The TEM image reveals quasi-spherical dots with a calculated particle size of 7.3 ± 1.2 nm – inset: particle size distribution shows that the particle size spans 5–9 nm; (B) AFM image of dCDs on a mica substrate. The height profile is 1.4 nm as shown in the inset; (C) room temperature absorbance and fluorescence spectra of a 50 μg mL^−1^ dCD dispersion. The UV-Vis absorption spectrum of dCDs (black curve) reveals three absorption bands centered at 295–350 nm, 370–450 nm and 590–690 nm. Following excitation at 405 nm, two fluorescence bands are observed at 370–500 nm and 640–730 nm (blue curve) while excitation at 640 nm reveals red fluorescence from 645–730 nm (red curve); (C, inset) dCD dispersion under white light (left) and UV light (*λ*_ex_ = 365 nm; right). The violet color is from the contribution of both blue and red fluorescence.

The dCDs were synthesized in a one-step microwave assisted-reaction using formamide and glutathione. Quasi-spherical dots were obtained following synthesis at 180 °C for a period of 5 minutes. Transmission electron microscopy (TEM) particle characterization (Fig. 1A) show that the dots are monodisperse with an average size of 7.3 ± 1.2 nm. Particle size statistics obtained for a large population of the dots reveals a Gaussian size distribution (Fig. 1A inset) ranging from ∼5–9 nm. Atomic force microscopy (AFM) analysis confirms our TEM findings showing that the dCDs are indeed spherical. The dCDs remain well dispersed in water and do not display any significant agglomeration upon drying on the AFM mica substrate with an average height of 1.4 nm (Fig. 1B inset).

We characterized the optical properties of the purified dCDs, which form a colourless dispersion in water at a concentration of 50 μg mL^−1^. Following exposure to UV (*λ*_ex_ = 365 nm), a violet colour is observed due to the simultaneous contributions of blue and red fluorescence (Fig. 1C inset). UV-Vis absorbance spectroscopy reveals that the dCDs have three distinct absorption bands at 295–350 nm, 370–450 nm and 590–690 nm (Fig. 1C). The first band is assigned to the transition of C=C bonds while the second and third bands can be ascribed to the π → π* and n → π* transitions of the aromatic sp^2^ domains for the C=O, as well as the C=N/C=S bonds, respectively.^42–44^ Thus, the dCDs can be excited at multiple wavelengths in the blue and red regions of the spectrum. Following excitation at 405 nm, the dCDs exhibit two distinct fluorescence bands between 370–500 nm (blue component) and 645–730 nm (red component) as shown in Fig. 1C. Conversely, excitation at 640 nm only results in fluorescence in the region of 645–730 nm. The ability to selectively excite the dCDs at longer wavelengths is attractive for bioimaging applications due to greater tissue penetration and decreased scattering.^10,30,42,45–49^ Quantum yield measurements were carried out to evaluate the fluorescence efficiency of the dCDs. Following excitation at 405 nm, a cumulative quantum yield of 6.49% was obtained using an integrating sphere with individual values of 0.38% and 6.11% ascribed to the blue and red components, respectively. This is expected since the red fluorescence dominates its blue counterpart as shown in Fig. 1C. Colloidal dCDs remain optically stable over a span of 6 months (Fig. S1†) and their optical properties do not suffer any significant degradation. The unique dual-fluorescence properties have been attributed to a mechanism consisting of carbon core and surface molecular fluorescent states.^28^ Herein, the blue fluorescence stems from the core of the dCDs, while the red counterpart originates from the fluorophores covalently attached to the carbon core.^28,49^ As shown in Fig. S2,† the fluorescence stemming from the core states is excitation-dependent in the blue region of the spectrum while the excitation-independent red fluorescence originates from the molecular states. A control experiment (see Fig. S3†) was performed by separately reacting formamide and glutathione in the microwave at 180 °C for 5 min to study the optical properties of the individual precursors. No dual fluorescence was observed in this case with very weak fluorescence in the blue region of the spectrum. This highlights the necessity of both precursors for the formation of dCDs.

**Fig. 2:**
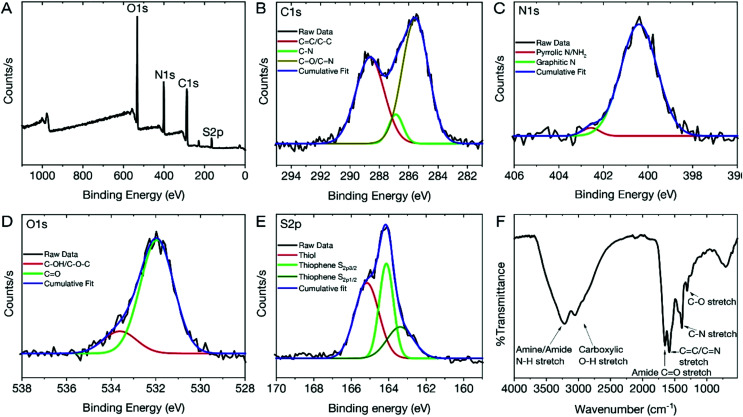
(A) XPS survey spectrum of the dCDs reveals 4 binding energies ascribed to C1s, N1s, O1s and S2p. Deconvoluted HR-XPS spectra of the binding energies are ascribed to (B) C1s at a maxima of 286.08 eV; (C) N1s at a maxima of 400.08 eV; (D) O1s at a maxima of 532.08 eV; (E) S2p at a maxima of 165.08 eV; (F) FT-IR spectrum of the dCDs revealing the presence of N–H and O–H surface groups along with amide and carbonyl stretches.

To assess the physico-chemical makeup of the dCDs and to understand the origins of their unique fluorescence properties, X-ray photoelectron spectroscopy (XPS) and Fourier Transform Infrared (FT-IR) analyses were carried out. The XPS survey spectrum of the dCDs (Fig. 2A) shows four binding energies at 532.08, 400.08, 286.08 and 165.08 eV corresponding to carbon (C1s), nitrogen (N1s), oxygen (O1s) and sulfur (S2s), respectively. This observation is in accordance with what is expected given that these four elements make up the chemical composition of glutathione or formamide. High-resolution XPS (HR-XPS) analysis of the C1s binding energies and subsequent deconvolution of the observed peak reveals three components at 288.68 eV, 286.89 eV and 285.58 eV ascribed to the presence of the C–C/C=C, C–O and the C=O/C=N functional groups, respectively (Fig. 2B). Similarly, the HR-XPS N1s spectrum (Fig. 2C) can be deconvoluted resulting in binding energies at 402.71 and 400.58 eV, which represents NH_2_/pyrrolic N, as well as graphitic N, respectively. For O1s (Fig. 2D), the HR-XPS spectrum shows the presence of C–OH/C–OC and C=O functional groups with deconvoluted binding energies observed at 533.26 and 532.02 eV, respectively. Lastly, for S2p (Fig. 2E), three prominent binding energies are noted at 165.51, 164.14 and 163.38 eV; the former is attributed to the thiol functional groups on the dCDs while the latter is assigned to the presence of thiophene groups. The data confirm the presence of C=O, C=N and C=S, which are believed to be found in the conjugated network responsible for the red absorption/emission shown in Fig. 1C.^42,43^ The dCDs are comprised of 53.1% carbon, 26.1% oxygen, 17.4% nitrogen and 3.4% sulfur as determined using HR-XPS spectra. Our XPS assignments are corroborated by FT-IR analysis (Fig. 2F). The presence of a broad peak from 3000–3500 cm^−1^ is assigned to the N–H and O–H stretching vibrations originating from hydroxyl and amine groups, while stretches at 1645 and 1583 cm^−1^ reveal the presence of C=O of an amide and of C=C/C=N bonds, respectively. The presence of an amide bond is further confirmed *via* observation of stretches at 1386 cm^−1^ and 1307 cm^−1^, which are attributed to the C–N amide bonds. The thiol stretch is not observed in the FT-IR spectrum, which is expected since this stretching vibration is characteristically weak in intensity.

### Temperature-dependent fluorescence

**Fig. 3:**
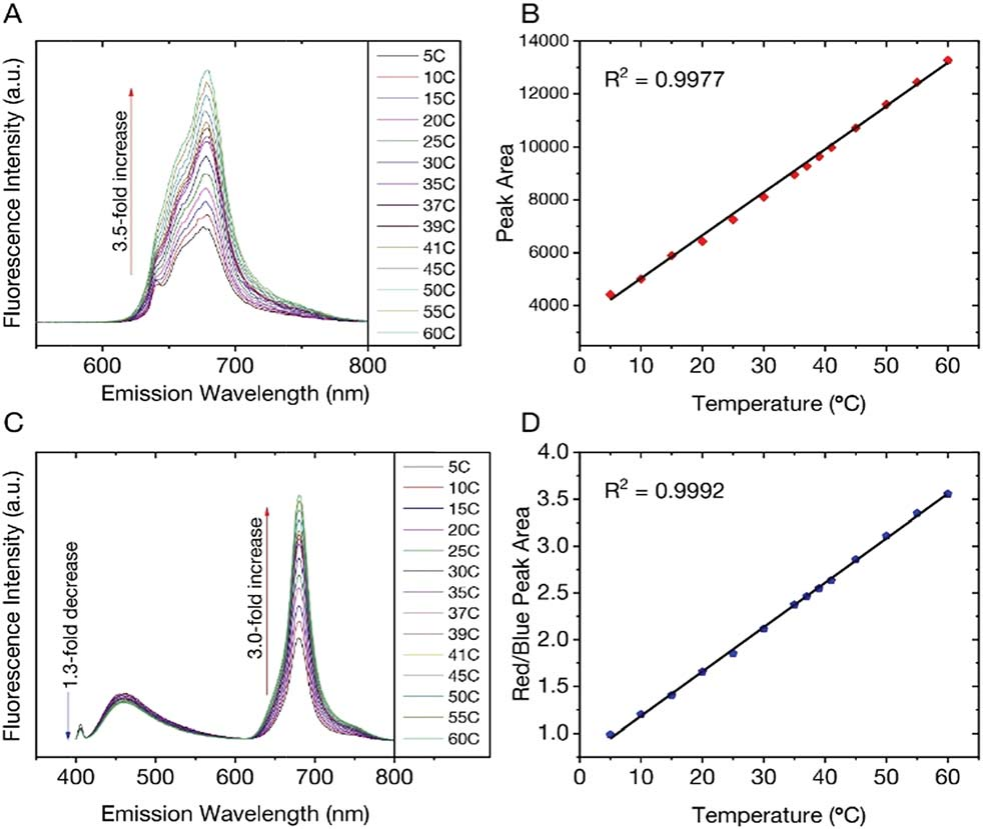
(A) Excitation at 640 nm yields a 3.5-fold increase in fluorescence intensity and the corresponding integrated area is plotted in (B) showing a linear response over the range of 5–60 °C; (C) changes in the fluorescence spectra of the dCDs (*λ*_ex_ = 405 nm) as a function of temperature over the entire range. A 1.3-fold decrease is noted for the blue fluorescence in contrast to the 3-fold increase for the red counterpart; (D) the ratio of the integrated areas of the red and blue fluorescence components are plotted as a function of temperature showing a linear increase over the entire temperature range.

We found that the fluorescent properties of aqueous colloidal dispersions of dCDs are temperature-dependent. Following excitation at 640 nm, the fluorescence intensity and integrated area increase over the range of 5–60 °C by a factor of 3.5 (Fig. 3A). As shown in Fig. 3B, a linear response (*R*^2^ = 0.999) is observed over the entire analysis range and the temperature sensitivity was determined to be as high as 3.71% °C^−1^. This value is comparable with various other polymer- and quantum dot-based nanothermometers.^50^

The temperature-dependent fluorescence was also studied following excitation at 405 nm. Interestingly, the blue and red fluorescence bands are not equally sensitive to the change in temperature. With increasing temperature, the fluorescence intensity (and corresponding integrated area under the curve) of the blue component shows a very slight decrease in contrast to the red component, which significantly increases (Fig. 3C). These observations are noted over the range of 5–60 °C where the blue emission decreases by a factor of 1.3 in contrast to the red emission, which increases by a factor of 3.0. Using eqn (1), a red-to-blue fluorescence ratio is obtained for every analysis temperature.1
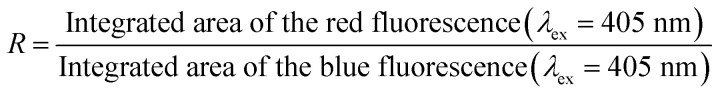


As shown in Fig. 3D, the ratio of red to blue fluorescence increases with temperature and a highly linear response is observed with an *R*^2^ = 0.998. These analyses were repeated in triplicate on 3 unique samples and the linear plot reflects the average of these measurements, which have small deviations at each temperature. The thermal sensitivity of the dCDs, over the entire temperature range, varied from 1.33–4.81% °C^−1^, which is an improvement over previously reported carbon dot nanothermometry systems^16,23^ and other dual-emitting nanomaterials such as quantum dots and metal organic frameworks-dye composites.^51–53^ The thermal resolution of the dCDs was calculated to be 0.048 K^−1^ indicating that it is indeed possible to measure small thermal changes. Our system is unique in comparison to other carbon dot systems since the dual fluorescence originates from the same probe and does not require the addition of a second fluorescent probe. It should be noted that the fluorescence response of the dCDs, following a thermal event, is quick thus making it an ideal candidate for real-time temperature monitoring. As shown in Fig. S4,† the fluorescence ratio stabilizes after only 2 min, which is the time required for the entire volume of the cuvette to reach thermal equilibrium.

The reversibility and stability of the temperature-dependent fluorescence of dCDs were evaluated through thermal cycling experiments (Fig. S5†). The thermal reversibility of dCDs was assessed by measuring recovery through multiple heating and cooling cycles from 5 to 60 °C. The results show that the initial fluorescence intensity does not significantly change following multiple heating and cooling cycles indicating their ability to recover following a thermal event.

### Cell uptake and cytotoxicity of dCDs

**Fig. 4:**
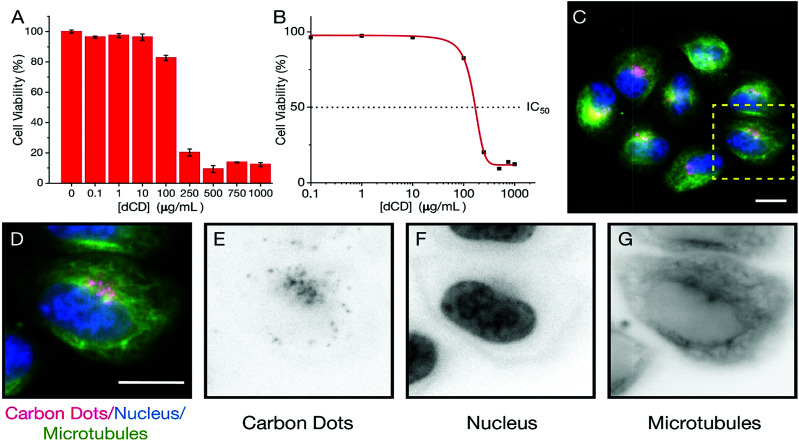
The viability and localization of dCDs in HeLa cells 48 h after treatment is shown. (A) The bar graph shows the proportion of live HeLa cells treated with different concentrations of dCDs as indicated on the *x*-axis; (B) an IC_50_ graph using log-scale shows HeLa cancer cell viability, which was measured to be 147.8 μg mL^−1^ (dotted line); (C) fluorescence microscopy image showing dCD-treated HeLa cancer cells co-stained with Hoechst to label DNA (blue) and Tubulin Tracker™ Green to label microtubules (green). The scale bar corresponds to a length of 10 μm; (D) a magnified image of a single cell (dashed box in C) shows the nucleus (DNA; blue), microtubules (green) and red-emitting dCDs. Inverted greyscale images show the (E) carbon dots, (F) nucleus and (G) microtubules.

We extended our study of the temperature-dependent optical properties of dCDs in cultured mammalian cells to validate their use in a model biological system. Prior to evaluating their temperature-sensing capabilities, the cytotoxicity of dCDs was assessed in HeLa cervical cancer cells. As shown in Fig. 4A, viability assays were performed using increasing concentrations of dCDs (from 0–1000 μg mL^−1^) for 48 hours. Cells remained viable up to a concentration of 100 μg mL^−1^ of the dCDs with an IC_50_ value (*i.e.* concentration at which 50% of the population remains) of 147.8 μg mL^−1^ (Fig. 4B). This suggests that the dCDs have low cytotoxicity and are biocompatible.

To determine their fluorescent properties and subcellular location, HeLa cells were treated with 100 μg mL^−1^ of dCDs for 24 hours to allow for cellular uptake and then imaged using fluorescence microscopy at 640 nm (Fig. 4C–G). The cells survived and maintained their integrity over 24 hours in support of the dCDs' low cytotoxicity (see Fig. S6†). Interestingly, the dCDs accumulated in regions that likely correspond to the endomembrane network. This network is typically found surrounding and adjacent to the nucleus (shown in blue; Fig. 4D–F). Considering that the localization of the dCDs is specific to this perinuclear region (endomembrane network), the fluorescence remains unaffected by any variation in pH. In HeLa cells, the pH of the endomembrane network is approximately 6.6, while that of the cytoplasm is typically at 7.0–7.4.^54^ We have studied the dependence of fluorescence on the pH of the medium (Fig. S7†) and it was observed that at pH values between 4 and 8, there are no significant changes to the fluorescence ratios for the dCDs. As such, the reported ratios measured in both the cuvette and intracellular models are reliable.

### Intracellular temperature-dependent fluorescence

**Fig. 5:**
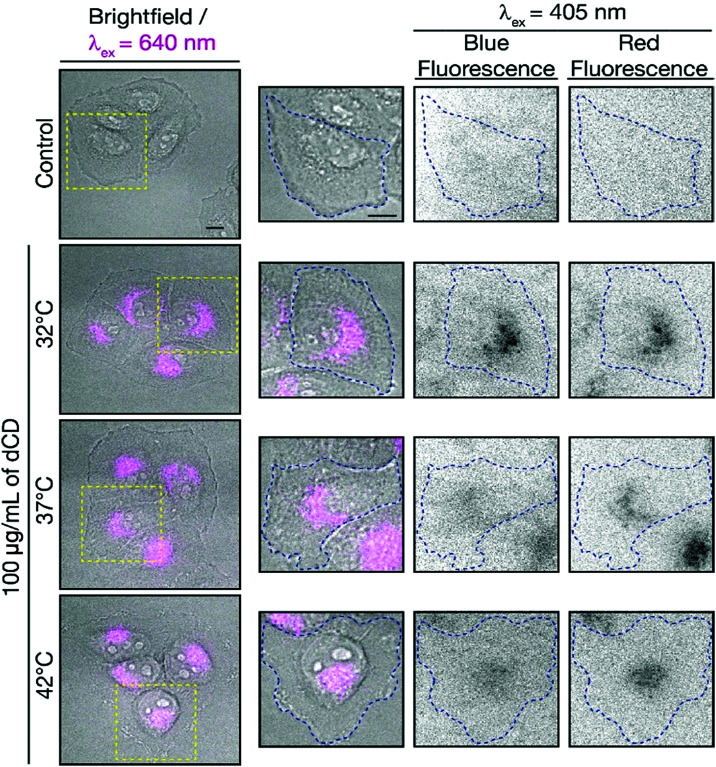
Fluorescence microscopy images of dCD-treated HeLa cells. Fluorescence signals from the dCDs (*λ*_ex_ = 640 nm; left and 405 nm; right) are shown for cells incubated at the different temperatures as indicated. The scale bars represent a length of 10 μm. The red-to-blue fluorescence ratios are 1.8 at 32 °C, 2.0 at 37 °C and 2.3 at 42 °C. The control shows untreated HeLa cells at 42 °C with no fluorescence signal as expected.

To determine if the dCDs could sense temperature changes inside the cells, *in vitro* thermal sensing using both intensity and ratiometric approaches were performed. HeLa cells treated with dCDs were allowed to equilibrate at 32, 37 and 42 °C (see Fig. 5, and S8† for controls at each temperature). Excitation at 640 nm allows us to selectively monitor the red fluorescence of the dCDs in cells. As shown in Fig. S9,† the thermal changes did not correlate with a change in intensity (*λ*_ex_ = 640 nm). This could be due to changes in intracellular concentration or localization of the dCDs at higher temperature. Thus, simply relying on changes in fluorescence intensity does lead to accurate intracellular thermal sensing.

In contrast, we do not have these limitations using the ratiometric approach. The dCDs maintain dual blue and red fluorescence in cells following excitation at 405 nm, as previously observed for the colloidal dispersions (Fig. 5). The red-to-blue ratio increases with increasing temperature, with values of 1.8 at 32 °C, 2.0 at 37 °C and 2.3 at 42 °C. Strikingly, these values are comparable to those observed in the cuvette model suggesting that the ratiometric relationship of the red-to-blue fluorescence of the dCDs is maintained in cells, as shown in Fig. S10.† This highlights the advantage of ratiometric temperature sensing in the development of fluorescent nanothermometry probes. Regardless of the amount of dCDs taken up by the cells, which can be affected by various factors such as confluency, the relative red-to-blue emission ratio remains unaffected and is not concentration dependent. Lastly, the dCDs have shown fluorescence reversibility with respect to changes in intracellular temperature. Following incubation in HeLa cells, they were subjected to a heating/cooling cycle from 32 → 42 → 32 °C. Rapid temperature changes were induced through the replacement of the cell media at the respective temperatures. Following analysis of an excess of 30 cells at each thermal event, the fluorescence properties remained unaffected with corresponding ratios of 1.5 → 2.2 → 1.7. This highlights the robustness of the proposed dCD-nanothermometer and these findings further demonstrate the fluorescence reversibility as shown in Fig. S5.†

## Conclusion

In this study, we describe the synthesis and physico-chemical properties of biocompatible dual-fluorescing carbon dots *via* the microwave reaction of formamide and glutathione. The water dispersible dCDs possess temperature-sensitive and reversible fluorescence. They show linear responses to the change in temperature from 5 to 60 °C in a cuvette model determined by intensity-based and ratiometric temperature sensing methods. These results translate into an *in vitro* cell model, where the ratiometric change in fluorescence was also observed in cells incubated at different temperatures. Unlike intensity-based measurements, temperature measurements using the ratiometric approach are not impacted by changes in dCD concentration, which could vary due to altered cellular uptake depending on cell density and/or temperature. With its facile synthesis, unique optical properties and biocompatibility, these dCDs are good candidates for *in vivo* nanothermometry in biological systems. Furthermore, they could be explored for bioimaging applications in the future.

## Experimental section

### Chemicals and reagents

Formamide (≥99.5%) and reduced l-glutathione (≥98.0%) were purchased from Thermo Scientific. Phosphate buffer solution (PBS, 1×) and Dulbecco's modified Eagle medium (DMEM) were purchased from Wisent. HyClone™ Calf Serum and Tubulin Tracker Green™ were purchased from Thermo Scientific. The WST-8 Cell Proliferation Assay Kit was purchased from Cayman Chemical. All reagents were used without further modification or purification.

### Synthesis of dual-fluorescing CDs (dCDs)

The dCDs were synthesized using a modified microwave-mediated one-step reaction with glutathione and formamide as previously reported.^36^ Briefly, a 20 mL solution of 0.1 M glutathione in formamide was prepared. The mixture was sonicated for 15 min until it changed from a cloudy to a clear homogeneous solution. The solution was then transferred to a 35 mL microwave reaction vial and heated to 180 °C for 5 min. Once cooled, the dispersion was dialyzed using a cellulose ester membrane dialysis membrane (molecular weight cut-off = 3.5–5.0 kDa) to remove unreacted materials and fluorophores. The sample was dialyzed for over five days with the water changed twice a day. Following this, the sample was passed through a 0.2 μm nylon filter to remove any aggregates. Subsequently, the samples were washed twice with ethanol and twice with acetone (1 : 10, sample : solvent volume ratio) to remove any remaining impurities. After each wash, the precipitate was collected by centrifugation at 10 000 × *g* for 10 min. The resulting material was dried in the oven at 70 °C overnight and resuspended in water.

### Characterization techniques

For Transmission Electron Microscopy (TEM), CDs were dispersed in water at a concentration of 5.0 mg mL^−1^. Grids were prepared by pipetting 2 μL of the dCD dispersion onto a 200 mesh Formvar/carbon coated copper grid (3 mm in diameter) followed by evaporation of the solvent. The TEM images were collected using a Jeol JEM-2100F microscope operating at 100 kV. Images were processed and the carbon dot sizes were determined using Fiji imaging software.^47^ UV-visible absorption spectra were acquired from 200–800 nm on a Cary 5 Series UV-Vis-NIR Spectrophotometer (Agilent Technologies) using a 1 cm quartz cuvette. A 5.0 nm bandwidth and wavelength changeover at 450 nm were used for analysis. Data was processed using Cary Eclipse software. Quantum yield values were acquired on an FLS920 Fluorescence Spectrometer (Edinburgh Instruments) with an integrating sphere using a 1 cm quartz cuvette. The excitation and the emission slits were set to a width of 5 nm. The excitation wavelength was set to 405 nm and the spectra from 300–800 nm were collected. Scans were done in triplicates with a dwell time of 0.2 s. Data was processed using F900 Software. Fluorescence spectra were acquired using a Cary Eclipse fluorescence spectrophotometer (Agilent Technologies). Spectra were acquired in a 1 cm quartz cuvette at *λ*_ex_ = 360–660 nm (5 nm intervals). The excitation and emission slits were set to a width of 5 nm with a PMT voltage at 600 V. All data were processed using Cary Eclipse software. To obtain fluorescence spectra at various temperatures, the Cary Single Cell Peltier Accessory (Agilent Technologies) was used to adjust the temperature from 5 °C to 60 °C. Fourier-Transform Infrared Spectroscopy (FT-IR) spectra were collected using a Thermo Scientific Nicolet iS5 equipped with an iD5 ATR accessory. Spectra were collected using 30 scans with a resolution of 0.4 cm^−1^, a gain of 1, an optical velocity 0.4747 and an aperture setting of 100. Data was processed using Omnic 9 software. X-ray Photoelectron Spectroscopy (XPS) spectra of the dCDs were acquired using a Thermo Scientific K-Alpha X-ray Photoelectron Spectrometer. Each analysis was carried out in triplicate with 10 runs for each scan. The averages were plotted for both the survey and high-resolution scans. The stability of the dCDs (50 μg mL^−1^) was evaluated by measuring the fluorescence intensities from the same stock solution of dCDs once a month over a span of 6 months. Fluorescence intensity (*λ*_ex_ = 640 nm) centered around 680 nm was collected and used for the assessment.

### Cell culture and WST-8 assays

HeLa cells were cultured in DMEM containing 10% calf serum and maintained in a humidified incubator set to 37 °C with 5% CO_2_. To assess cell viability, HeLa cells were plated in 96-well plates at 20% confluency and cultured overnight. The cytotoxicity of the dCDs was assessed *via* WST-8 cell proliferation assays (Cayman Chemical). The cells were treated with dCDs at varying concentrations (0 to 1000 g mL^−1^) and incubated for 48 hours. Subsequently, the WST-8 reagent was added for 4 hours. The optical density (OD) values were measured using the TECAN 200 PRO plate reader at a wavelength of 490 nm. Cell viability was measured as a ratio of the signal of treated cells *vs.* control:2
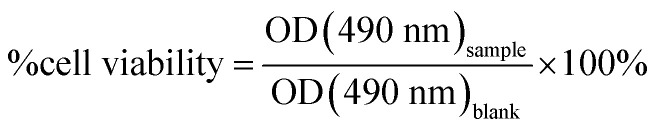


All measurements were repeated in triplicate and the means were plotted with standard deviation.

### Cellular imaging

HeLa cells were imaged after treatment with the dCDs. Cells were plated in Nunc™ Lab-Tek II 4-well chambered microslides (Thermo Scientific™) and left overnight to adhere. The dCDs were added to cells in media at a final concentration of 100 μg mL^−1^ for 24 hours. To determine the localization of dCDs, HeLa cells were treated with TubulinTracker Green Reagent (Molecular Probes) at a final concentration of 250 nM as per manufacturer's instructions to stain microtubules and Hoechst 33342 dye (Thermo Scientific™) at a final concentration of 75 nM to stain the DNA. The cells were then placed in the incubator at 37 °C with 5% CO_2_ for 30 minutes after which the media was replaced. Imaging was performed using the Nikon-TIE inverted epifluorescence microscope with Lambda XL LED light sources using the 60×/1.4 oil objective, a Piezo Z stage (ASI), a Photometrics Evolve 512 EMCCD camera and Elements 4.0 acquisition software (Nikon). Images were captured as 0.5 μm *Z*-stacks and converted into maximum intensity *Z*-stack projections using FIJI (NIH).

### Intracellular nanothermometry

To measure the nanothermometric properties of the dCDs, HeLa cells were prepared as described above. In each Lab-Tek II chamber, three wells containing Hela cells were treated with 100 μg mL^−1^ of dCDs and placed at 32 °C, 37 °C or 42 °C for 2 hours prior to imaging (*n*_i_ = 30 cells). The controls were subjected to the same conditions, except without addition of the dCDs. After 2 hours, the wells were placed into an INU-TiZ-F1 chamber (MadCityLabs) with 5% CO_2_ and heated at their respective experimental temperature for imaging, which was mounted on the stage on the inverted microscope. Imaging was carried out using the same microscope described above. Excitation at 405 or 640 nm was used to capture emission at 460 nm using a 400–600 nm bandpass (Chroma CT500/200bp) filter, or at 680 nm using a 610 nm longpass (Chroma AT610lp) filter, respectively. Images were acquired as 0.5 μm *Z*-stacks using Elements 4.0 software (Nikon) and converted into maximum intensity *Z*-stack projections using FIJI (NIH). Background intensities were subtracted using the control. *In vitro* intracellular temperature was monitored following a thermal cycling regime to assess the reversibility of the fluorescence properties. Initially, the dCD-treated cells were incubated at 32 °C. Then, the temperature was rapidly cycled by replacing the media with media already equilibrated at 42 °C. Finally, the temperature was cycled down to 32 °C once again by replacing the media. The red-to-blue emission ratios were calculated at each temperature.

## Conflicts of interest

There are no conflicts to declare.

## Supplementary Material

NA-001-C8NA00255J-s001
