# Supplementary material for: Intracellular ratiometric temperature sensing using fluorescent carbon dots
Source: Nanoscale Adv. 2018 Dec 10;1(1):105–13. doi: 10.1039/c8na00255j (PMC9473198; doi:10.1039/c8na00255j)
Supplement: NA-001-C8NA00255J-s001 [file NA-001-C8NA00255J-s001.pdf]

Electronic Supplementary Material (ESI) for Nanoscale Advances.
This journal is © The Royal Society of Chemistry 2018

Supporting Information

Intracellular Ratiometric Temperature Sensing Using Fluorescent Carbon Dots

Jun-Ray Macairan,[1] Dilan Jaunky,[2] Alisa Piekny,[2] Rafik Naccache[1,*]

[1] *Department of Chemistry and Biochemistry and Center for NanoScience Research, Concordia University, Montreal, QC, Canada, H4B 1R6*

[2] *Department of Biology and Center for Cellular Microscopy and Cell Imaging, Concordia University, Montreal, QC, Canada, H4B 1R6*

* = Corresponding author (email: rafik.naccache@concordia.ca)

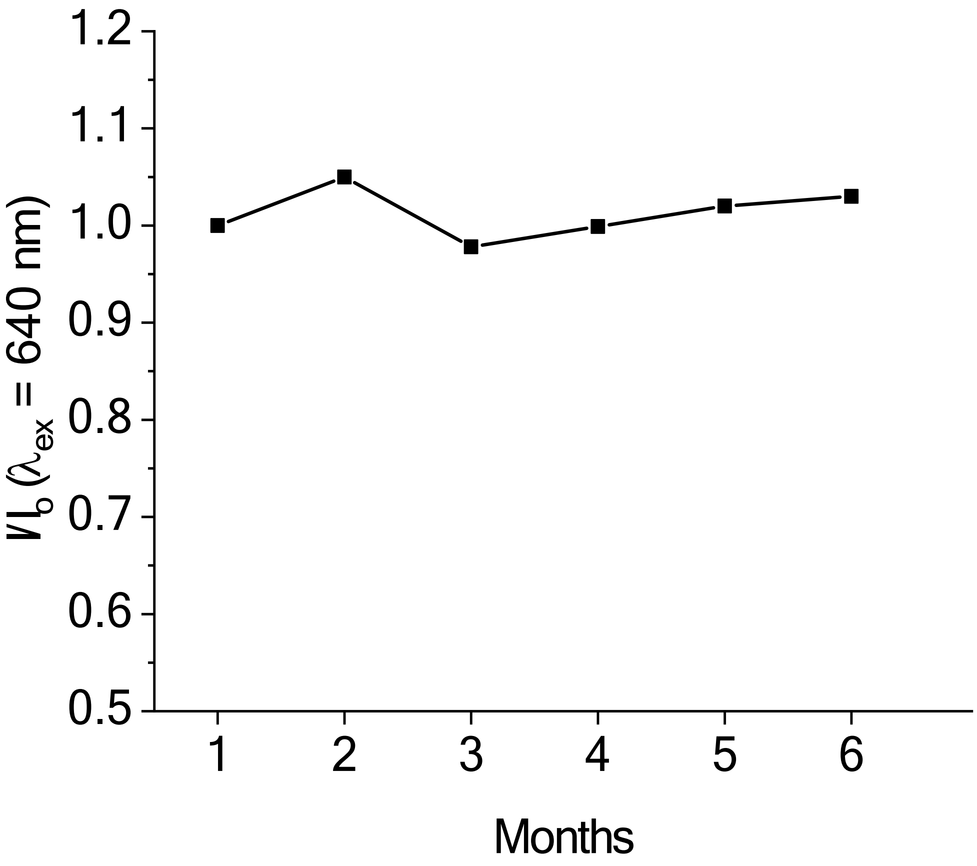

Figure S1. The stability of the dCDs was assessed by measuring the fluorescence at 683 nm (λ_{ex} = 640 nm) once a month over a span of 6 months. No significant changes were observed over this time span.

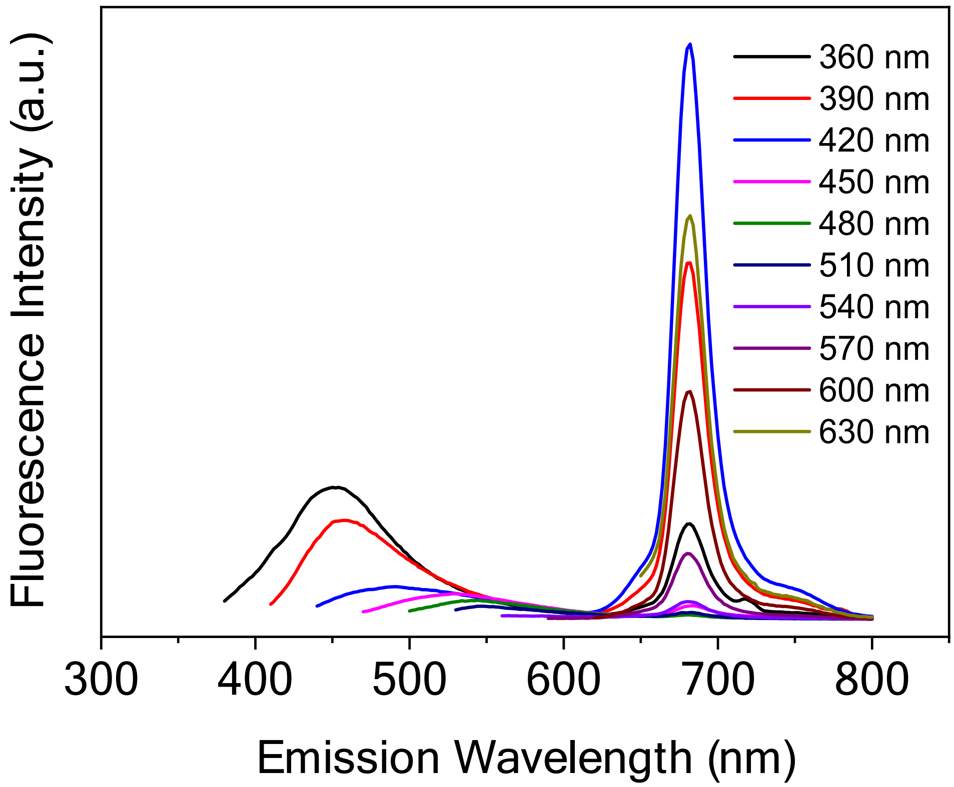

Figure S2. Fluorescence spectrum of a 50 μg/mL dCD dispersion highlighting the carbon core and molecule state fluorescence observed for dCDs. The excitation-dependent blue emission stems from the carbon core states and the excitation-independent red emission originates from the surface molecular states.

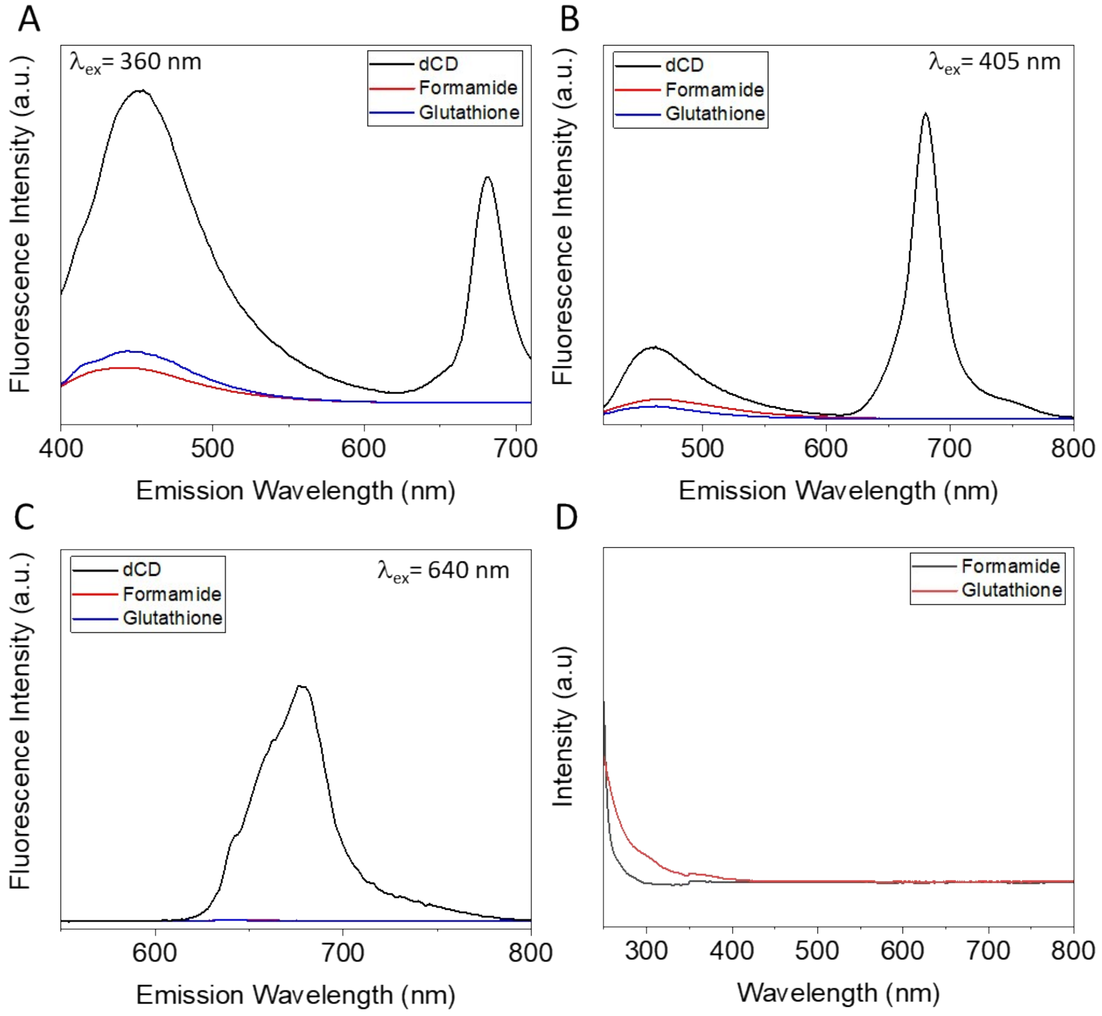

Figure S3. (A-C) Fluorescence spectra of formamide and glutathione (dissolved in water) reacted separately in the microwave for 5 min at 180 °C. Weak intensity fluorescence peaks are observed in comparison to dCDs. Moreover, these species possess a single fluorescence signal only in the blue region of the spectrum. Comparisons were carried out based on a concentration of 50 μg/mL of dCDs and a fivefold concentration of 250 μg/mL microwaved-formamide and 250 μg/mL microwaved-glutathione; (D) the absorbance spectra of formamide and glutathione, following microwave synthesis, show no significant absorption bands and no similarities to the absorbance spectrum of dCDs. These spectra highlight the importance of the two precursors to form dual-fluorescing carbon dots, something which cannot be achieved using a single precursor. In addition, it's noted that formamide and glutathione yield no fluorescence and absorbance signals in the absence of microwave heating.

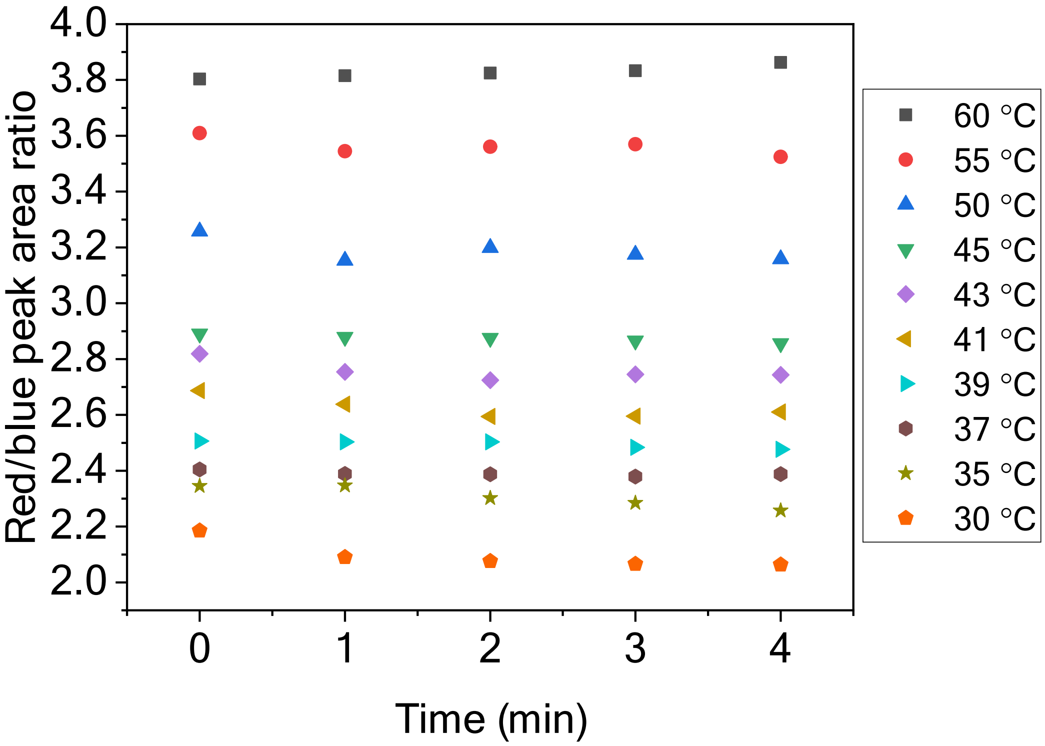

Figure S4. Time evolution of the fluorescence response at decreasing temperatures. The fluorescence was measured starting at 60 °C and then quickly cooled to a lower temperature as shown above. Each time point corresponds to the time elapsed once the single cell Peltier reading reached the desired temperature. The cooling time between temperatures was measured to be approximately 10-15 seconds. Plotted data suggests that the dCDs provide stabilized temperature readings after 2 minutes noting that this is the time required for the entire cuvette volume to reach thermal equilibrium.

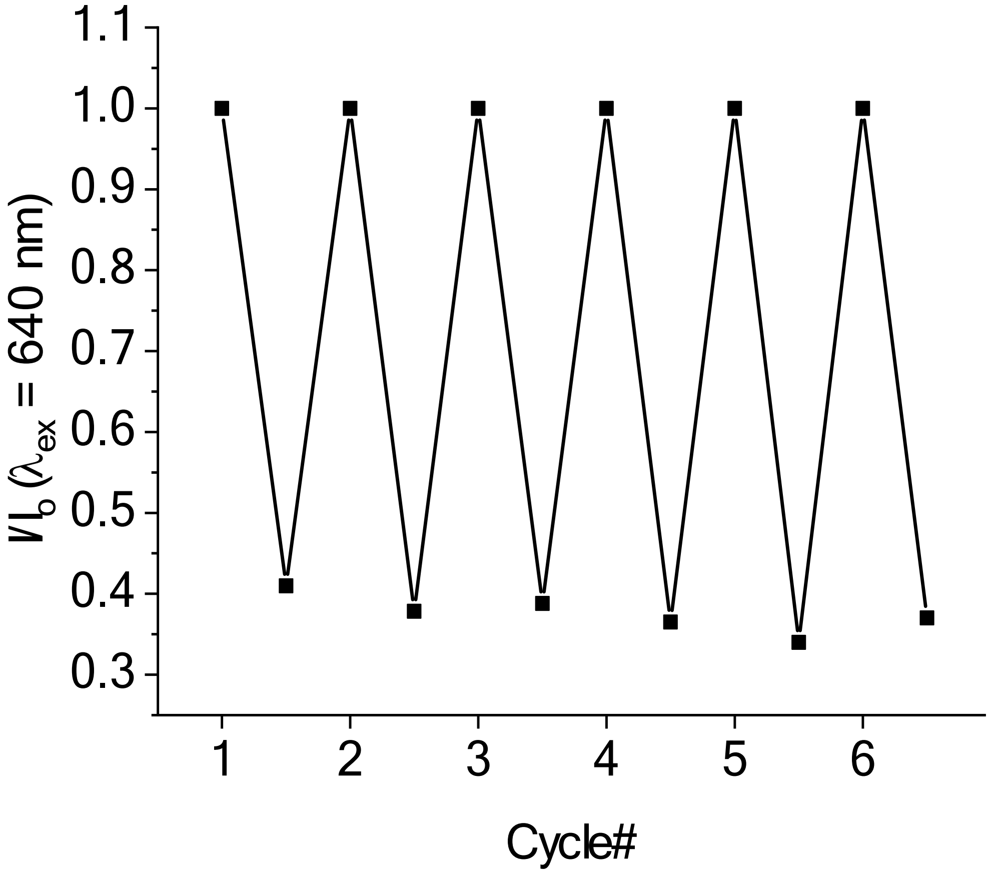

Figure S5. The reversibility of the dCDs was evaluated by measuring the intensity at 683 nm (λ_{ex} = 640 nm) between several heating/cooling cycles. The fluorescence was observed to recover following several heating and cooling cycles with no significant changes.

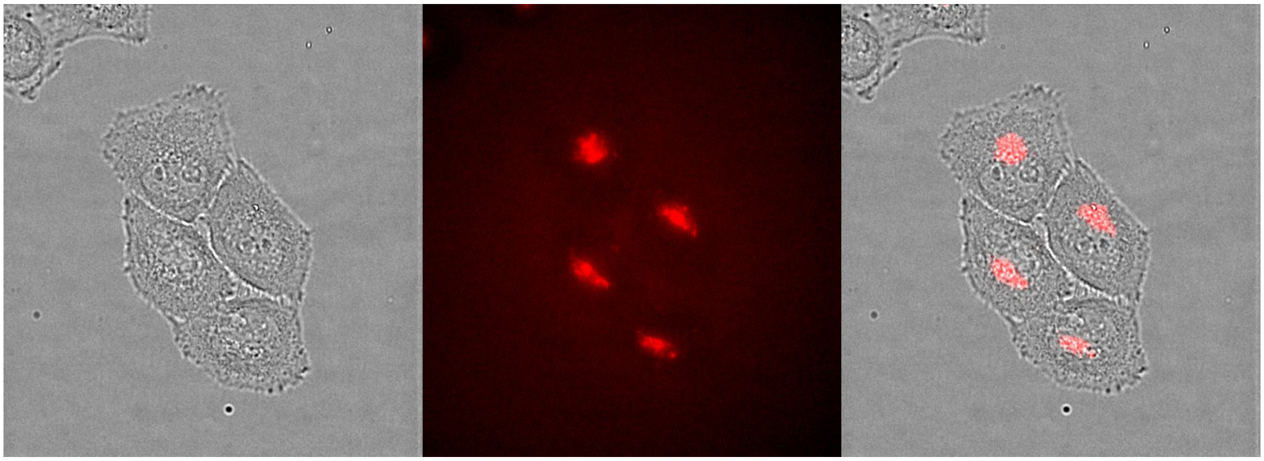

Figure S6. Fluorescence microscopy images of HeLa cells treated with 100 μg/mL of dCDs. From left to right: brightfield microscopy image of the treated HeLa cells, the fluorescence image (at $\lambda_{ex} = 640$ nm) of the treated HeLa cells showing the red fluorescence of the dCDs and the merged brightfield and fluorescence images. The integrity of the cell structure remained unaffected after 24 hours of dCD treatment.

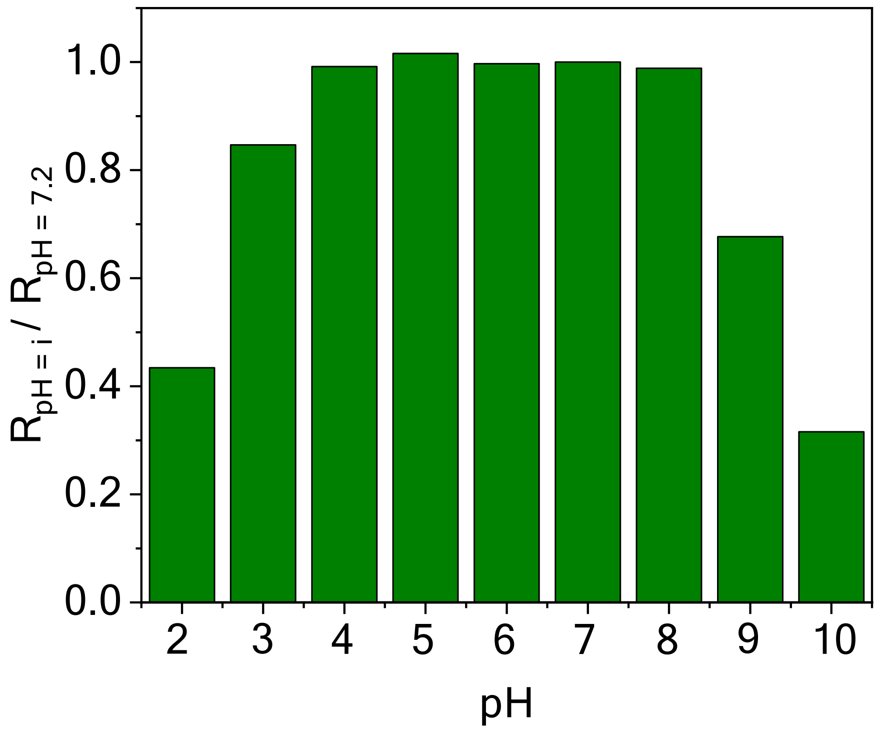

Figure S7. Relative red-to-blue fluorescence ratio of dCD colloidal dispersions at different pH levels. It is noted that the ratios do not change significantly between pH 4 - 8.

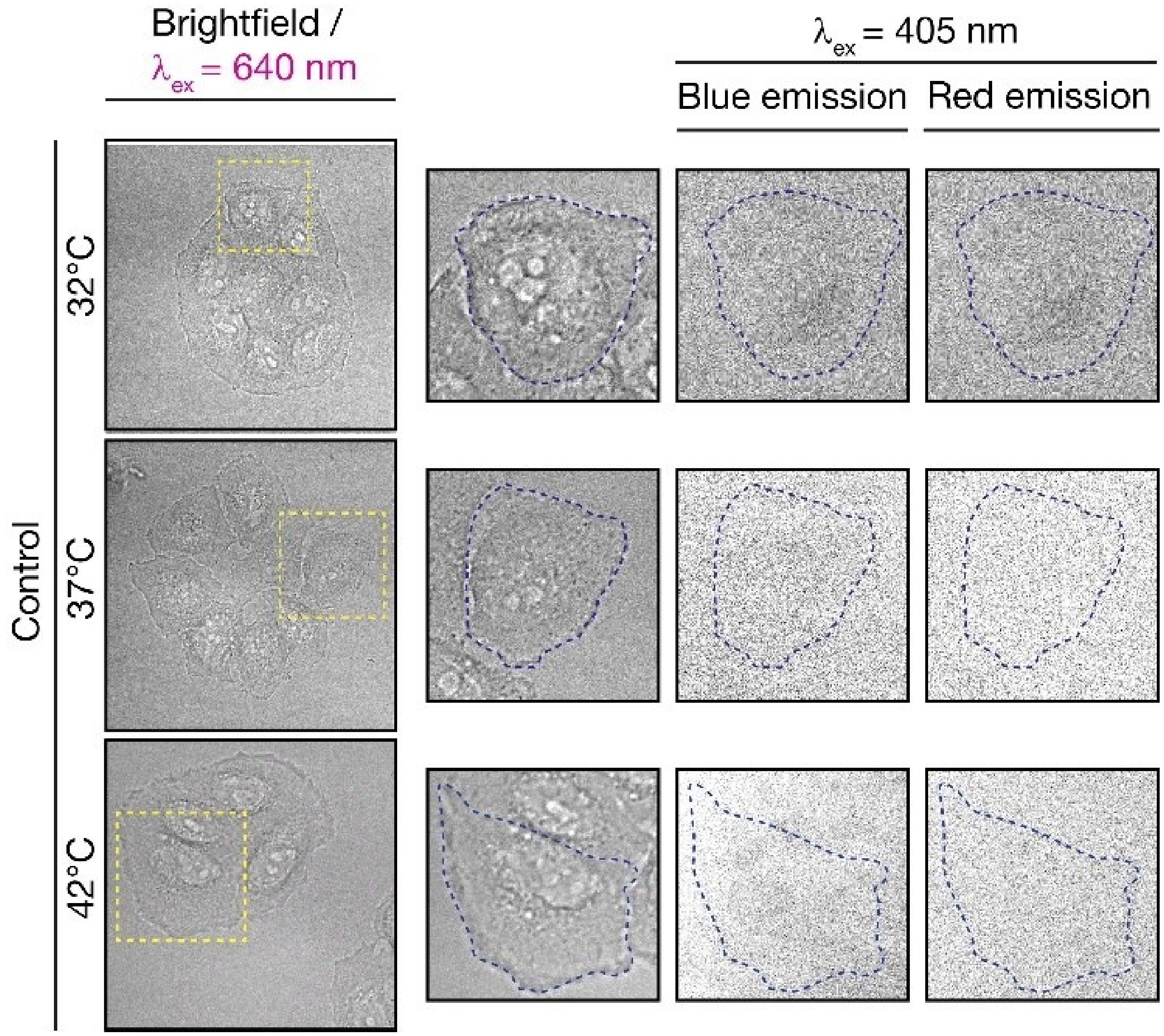

Figure S8. Cellular nanothermometry fluorescence microscopy controls, at various temperatures, using untreated HeLa cells (no dCDs). As expected, no fluorescence was observed in the endomembrane network.

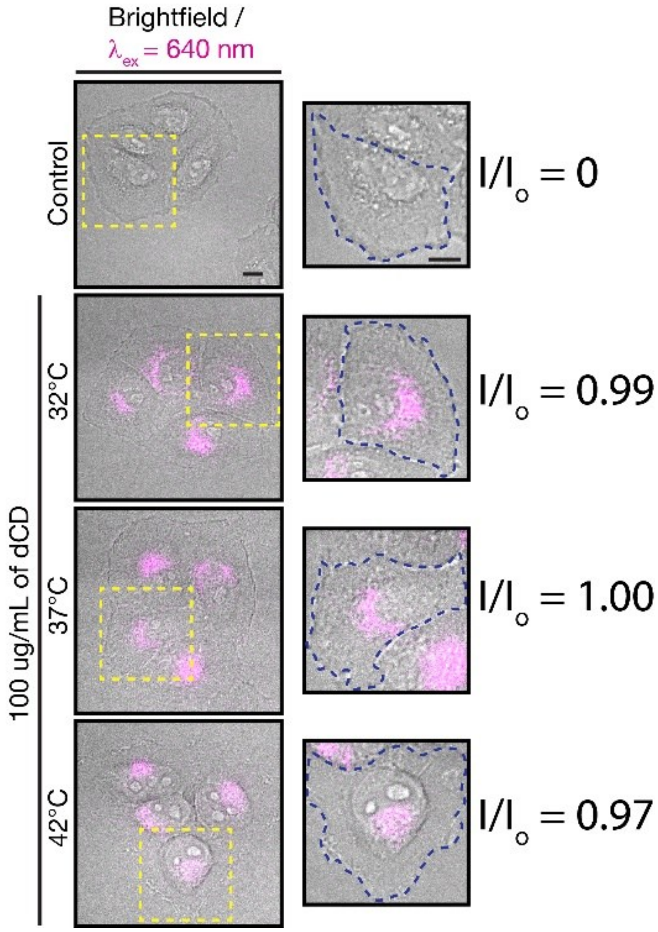

Figure S9. Overlay of brightfield and fluorescence images (λ_{ex} = 640 nm) of dCD-treated HeLa cells, at different temperatures. Temperature sensing solely using the red fluorescence signal is not feasible as the incubation temperature is likely affecting the cellular uptake generating a non-linear response.

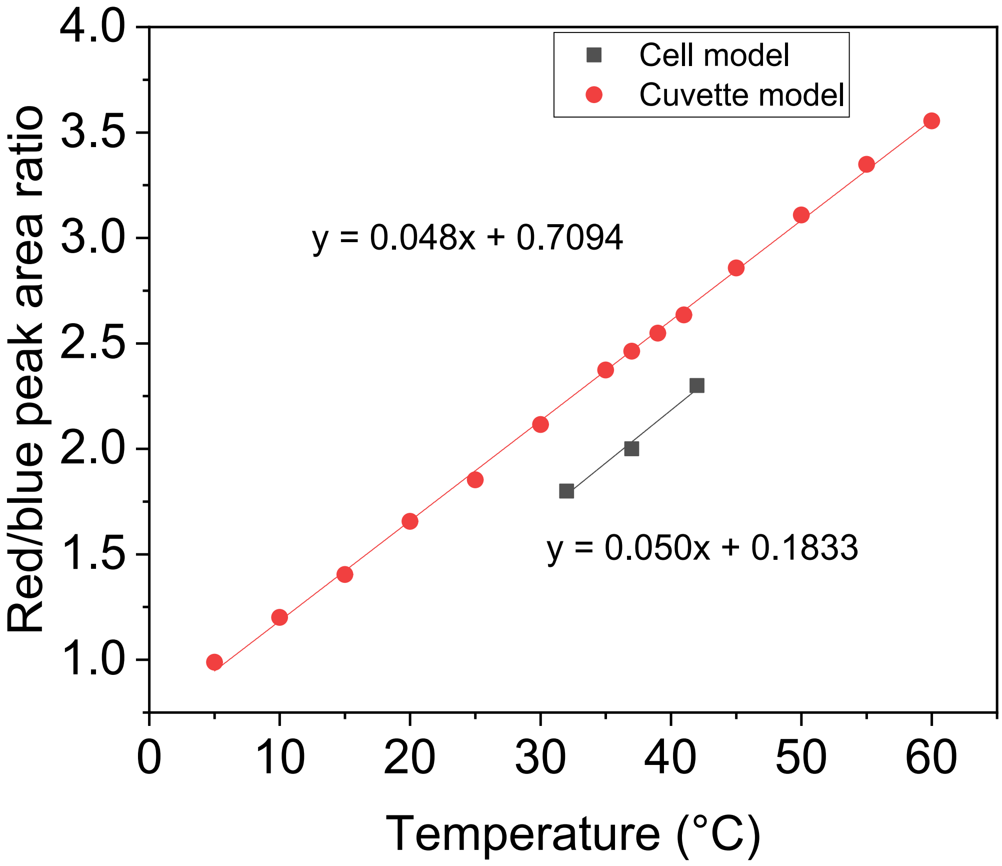

Figure S10. Changes in the red/blue fluorescence ratio of dCDs as a function of temperature (λ_{ex} = 405 nm) in both the cuvette model and intracellular models. Both demonstrate a linear ratiometric response relative to the change in temperature. Although their slopes are similar, the ratios are slightly lower in the cell model likely due to differences in the detection sensitivity between the epifluorescence microscope and the fluorimeter.
